# Placebo Cohorts in Phase-3 MS Treatment Trials – Predictors for On-Trial Disease Activity 1990-2010 Based on a Meta-Analysis and Individual Case Data

**DOI:** 10.1371/journal.pone.0050347

**Published:** 2012-11-29

**Authors:** Jan-Patrick Stellmann, Anneke Neuhaus, Lena Herich, Sven Schippling, Matthias Roeckel, Martin Daumer, Roland Martin, Christoph Heesen

**Affiliations:** 1 Institute for Neuroimmunology and Clinical MS Research (inims) and Department of Neurology, University Medical Center Hamburg-Eppendorf, Hamburg, Germany; 2 Sylvia Lawry Centre for Multiple Sclerosis Research, Munich, Germany; 3 Department of Medical Biometry and Epidemiology, University Medical Center Hamburg-Eppendorf, Hamburg, Germany; 4 Teva Pharma GmbH, Ulm/Mörfelden-Walldorf, Germany; 5 Neurology Clinic, Department of Clinical Neuroimmunology and Multiple Sclerosis Research, University Medical Center Zürich, Zürich, Switzerland; National Institutes of Health, United States of America

## Abstract

**Background:**

Annualized relapse rates (ARR) in the placebo cohorts of phase-3 randomized controlled trials (RCT) of new treatments for relapsing remitting multiple sclerosis (RRMS) have decreased substantially during the last two decades. The causes of these changes are not clear. We consider a better understanding of this phenomenon essential for valuing the effects of new drugs and by designing new trials.

**Objectives:**

To identify predictive factors of on-study ARR in early and recent MS trials.

**Methods:**

ARR, rate of relapse-free patients, trial start dates, baseline demographics, relapse definitions and the use of McDonald criteria were retrieved by literature research of the placebo cohorts from RRMS phase-3 trials. Predictors were estimated by univariate and multivariate regression analyses and random-effects meta-regression. In addition, regression models were calculated by the Sylvia Lawry Centre's (SLC), including individual case data from clinical trials performed until 2000. The most reliable meta-analytic results can be gained from pooled individual case data. In lack of this, random-effects meta-analyses are recommended.

**Results:**

Data from 12 published and one unpublished trial show a decrease of ARR from 1988 to 2012 (adjR^2^ = 0.807, p<0.0001). Regression models identified McDonald criteria followed by baseline mean age and the pre-study relapse rate as predictors of the ARR. The pooled individual case data (n = 505) confirmed a decrease of ARR over time. The pre-study relapse rate was the best predictor for on-study relapses. Lacking individual case data after implementation of the McDonald criteria excludes a direct comparison concerning McDonald criteria.

**Conclusion:**

Pre-study relapse rate was the best predictor for on-study relapse rate but failed to explain the decrease of the ARR over time alone. Higher age at baseline and the implementation of McDonald criteria were associated as well with a lowered relapse rate in the random-effects meta-regression. These findings need further clarification based on individual case data.

## Introduction

Relapses have been used widely as primary outcome in clinical phase-3 trials in relapsing-remitting Multiple Sclerosis (RRMS). Their future role in MS trial design is under discussion as annualized relapse rates (ARR) in the placebo cohorts of the 2010 published Fingolimod and Cladribine trials (ARR = 0.4 respectively 0.33) were substantially lower than in comparable trials of the 1990 s (e.g Interferon-ß-1a i.m. ARR = 0.9 and s.c. ARR = 1.28). [1]–[4] This observation leads to a major design problem for upcoming RRMS trials: with existing treatment options as comparator larger trials compensating for overall low event rates are necessary. Trials will become far more expensive and difficult to conduct. The reason, why placebo groups of recent trials show a less active disease course, is unclear.

A meta-analysis of demographic data and key points of study protocols as predictors for relapse rates during the last 20 years appears feasible.

In 2005, when reduction of relapse rates over time was not yet under discussion, Held et al. [5] used multiple regression models to identify predictors of the ARR. Data for this study were obtained from the Sylvia Lawry Centre for MS Research (SLC) individual case data base consisting of data from several placebo cohorts of phase-2 and 3 studies. The ARR of placebo patients in RCTs up to the year 1999 correlated well with the pre-study relapse rate and inversely with the disease duration at baseline. A possible change of the behaviour of placebo cohorts over time was not assessed by this approach. MRI variables at baseline did not show an additional predictive value on relapse and disability outcomes in RRMS populations. [6], [7] Inusah et al. were the first to analyse the reduction of the ARR over time (1981 till 2008) in placebo groups of phase-2 and 3 trials by univariate and multivariate regression analysis in 2010. They found a predictive value of lower baseline mean age and of the definition of relapses either with a minimal duration of 24 or 48 hours for higher ARR on study. [8] Their analysis was limited in including only ARR as disease activity criterion in the multivariate regression. Furthermore, they analysed RRMS as well as secondary-progressive MS (SPMS) cohorts in a heterogenous mixture of phase-2 and 3 studies, whereas recent large phase-3 trials were not included.

To the best of our knowledge, the phase-3 studies published till 2012 have not been included in a meta-analysis yet. Furthermore, a random-effects meta-regression has never been performed in this dataset. Random-effects meta-regression is recommended and widely used to analyse the heterogeneity of studies in a meta-analysis. [9]–[11] Heterogeneity derives from different trial designs or different demographic characteristics of study populations. As the variance of the ARR may reflect heterogeneity of studies, the above mentioned meta-regression methodology seems the most appropriate statistical method to evaluate different variables as possible moderators of this heterogeneity [9].

Based on this concept, we aimed at covering the widest possible time-frame of high quality phase-3 trials in RRMS to asses 1.) The predictive value of baseline demographic factors and 2.) The impact of the McDonald criteria (2001, revised 2005) for clinical disease activity.[12], [13] This is especially relevant as the actual revision of McDonald criteria has just been published allowing a MS diagnosis at the time of first symptoms. [14] By use of the SLC database, we wanted to 3.) test possible predictors from the meta-analysis based on individual case data.

## Methods

Our statistical analysis plan was designed to combine three possible ways of analysing predictors for relapse outcomes of randomized placebo controlled phase-3 clinical trials of relapsing-remitting MS: The results of (1) univariate and multivariate regression analyses and (2) a random-effects meta-analysis were compared. Additionally, we (3) evaluated cases in the open part of the SLC database. Exclusion of CIS, SPMS and primary-progressive MS (PPMS) patients seemed reasonable to avoid a bias towards MS courses with lower relapse activity. Phase-2 studies were not included in the analysis for the following reasons: relapses are not the usual primary outcome in MS phase-2 trials and there are no available data on relapse-free patients from most of these studies. Finally short durations between 6 and 12 months make estimates of the ARR less reliable. The ARR and the rate of relapse-free patients after 2 years (RRF) within the placebo cohorts were focussed as outcomes of interest. Based on the difficulties of relapse diagnosis and the on-going debate on the relevance of relapse numbers for prognosis the free-of-relapse criterion seems the more robust relapse outcome as it less influenced by number of relapses per patient and simply differentiates between missing or on-going relapse activity.

SLC has been collecting anonymised data from various data donors of natural history studies and randomized controlled clinical trials. The data it selves have been collected by the data donors with appropriate consent forms. Without explicit permission of the data donor even the data donor is anonymised.

### Selection of studies

**Figure 1:**
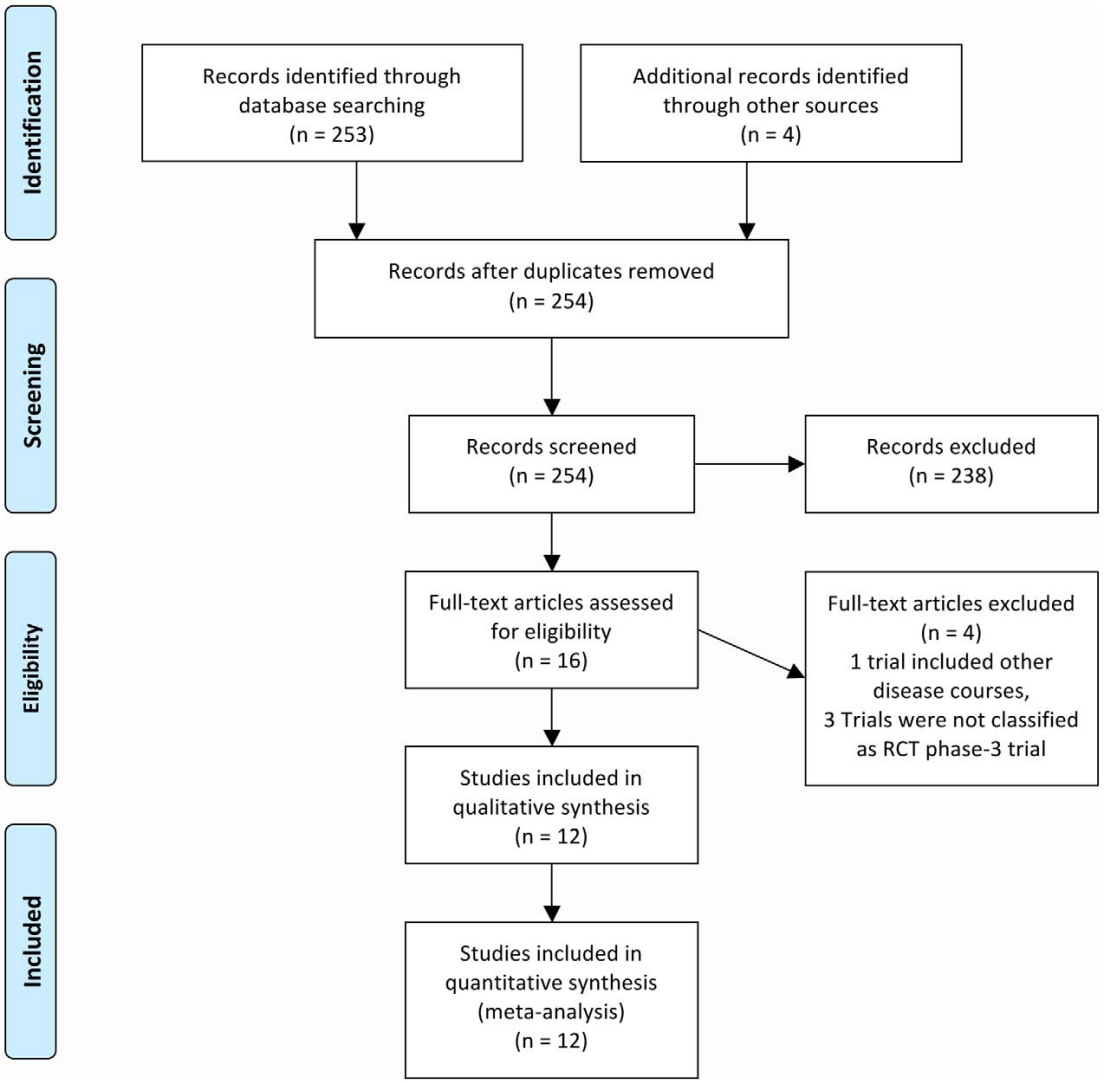
Flow chart: Study selection for meta-analyses. According to the PRISMA guidelines. [15].

At first, a comprehensive literature research within the Pubmed database was performed (last access in March 2012) by one reviewer (JPS) with the following keywords: ‘randomized placebo controlled trial in relapsing remitting multiple sclerosis’. The results of the electronic search (n = 253) was screened by headers and abstracts if available. Only full-length original English journal publications were reviewed to identify studies that met the following criteria (n = 11): (1) placebo-controlled double-blinded phase-3 trials in MS with a follow up of at least 18 months, (2) relapse related outcomes, (3) exclusion of SPMS, PPMS and CIS patients and (4) published between 1980 and March 2012. Record selection, exclusions and inclusion of studies according to the PRISMA guidelines are presented in figure 1.[15] JPS and CH. performed exclusions.

### Data extraction

From of the publications following data was extracted: the name of the first author, year of publication and number of patients in the placebo cohort; the trial start date, the ARR and the RRF; the baseline characteristics of these cohorts including mean age, mean disease duration, rate of females, mean pre-study relapse rate and mean EDSS; definition of disease duration (time since first symptoms or time since diagnosis), relapse definition (24 or 48 hours of minimal symptom duration) and whether the McDonald criteria were applied. Throughout this paper the ARR and the RRF are labelled as “outcomes” while trial start dates, baseline values and definitions are mentioned as variables. For all means the standard deviations (SD) were also extracted if published. Available confidence intervals or standard errors were converted to SD. Missing values and definitions were obtained from data on file by contacting authors and sponsors of the publications and trials. To avoid data copying mistakes, the final dataset was reviewed by two collaborators (JPS and SR).

### Statistical methods

Standard errors (SE) for the rate of relapse free patients were calculated as proposed by Gelmann and Hill.[16] Missing SD of ARR means were estimated under the assumption, that relapses follow a Poisson distribution. For all continuous variables a descriptive analysis with median, range, mean and SD was conducted. Scatter plots and regression analyses were used to determine if ARR and RRF did show a significant change over time defined by the trial start dates. Adjusted coefficients of determination (adjR^2^) between ARR/RRF and baseline values as well as between trial start date and baseline values were calculated. Besides these univariate models, multivariate regression models were applied as well. The included variables were selected by stepwise backward selection on base of the Akaike information criterion (AIC) that indicates the relative goodness of fit.[5], [17] All regression models were weighted for the number of patients.

Within random-effect meta-analyses the observed outcomes are understood as a random selection of the normally distributed outcomes.[18] Different designs and populations of these studies lead to an additional heterogeneity or variability of the true outcome. This can be taken into account by adding a random effect allowing an estimation of the heterogeneity with tau^2^ as the between-study variance. Within mixed effect models, variables may be added and analysed in their ability in reducing the heterogeneity of the pure random effects model.[18], [19] The reduction of tau^2^ indicates the association between outcome and variables.

Within our analysis we calculated I^2^ (proportion of heterogeneity among true effects of total variability) and tau^2^ (between study variance) for the pure random effects models. A detection of outliers was implemented. For the mixed-effect models we included each variable separately and calculated tau^2^ and its relative change compared to the pure random effect model as a measure of association between variable and outcome.[18] Finally, we modelled a mixed-effects analysis with the two variables which reduced the heterogeneity most effectively as recommended by Houwelingen et al.[19].

Besides descriptive statistics of the pooled placebo population of the SLC database (open part), Poisson regression models were used to examine the correlation of possible predictors with the ARR. Logistic regressions were calculated for the models concerning the outcome relapse free after 2 years. Date of study entry was used equivalent to the trial start date. To ensure the blinding of the SLC database and to avoid conclusions about a single study, date of study entry was constructed as ordinal variable with each rank including at least two studies. Other variables were selected according to the meta-regression analyses. The final multivariate models based on variable selection by AIC.

All analyses were performed with the open-source software R including the Hmisc and the metafor package.[18], [20], [21] The results of the random-/ mixed effect meta-analyses were revised with STATA®.

## Results

### Datasets

We identified 11 published trials [1]–[4], [22]–[28] that met our inclusion criteria. One further placebo cohort from an unpublished trial (ORIGIMS – A randomised, parallel group, placebo-controlled, double-blind phase-3 study of Bioferon® (interferon beta-1a) in the treatment of relapsing-remitting multiple sclerosis) could be added to the simple and meta-regression analyses from the SLC database, since the study sponsor had given its explicit consent to allow a separated re-analysis of the placebo cohort data. A dataset from a 13^th^ study could be added based on 2011 conference proceedings.[29], [30] The available data is presented in table 1. For all trials we could determine the RRF and calculate the corresponding SE. The ARR with corresponding SD was available from all studies except three. 9 Studies used the time since diagnosis as definition for the baseline disease duration while 4 trials used the time since first symptoms. These were labelled as missing for the analysis. All trials conducted before 2001 (n = 6) included only clinically definite MS-cases while the 7 most recent trials implemented the McDonald criteria (6 studies with the 2001 criteria, 1 study with the revised criteria from 2005). This dataset was used for the simple regression and the random-effects meta-regression. The SLC dataset of individual case data consisted of a pool of 505 placebo patients. The two datasets overlapped concerning pivotal trials from the 1990ies, the ORIGIMS data was only included in the first dataset and excluded from the individual case data due to blinding reasons. Individual case data from more recent trials was not available due to restrictive data sharing policies of the involved companies. Therefore, findings from the meta-regression could not be confirmed in a comparable individual case dataset.

**Table 1:** Overview of included RRMS phase-3 trials (n = 13).

			Outcomes after 2 years				Baseline data										Definitions		
Study	Start dates	N	RRF	SE	ARR	SD	Mean age	SD	Rate females	SD	Mean disease duration	SD	Pre-study mean relapse rate	SD	Mean EDSS	SD	Disease duration since	Minimal relapse duration	McDonald criteria used
'1993 INFB' [24]	1988	123	16%	3%	1.27	0.86	36.00	6.65	72%	4%	3.90	3.33	3.60	1.11	2.80	1.11	Diagnosis	24	no
'1996 Jacobs' [3]	1990	143	26%	4%	0.90	0.82	36.90	7.65	72%	4%	6.40	5.86	1.20	0.60	2.30	0.84	Diagnosis	48	no
'1995 Johnson' [25]	1991	126	27%	4%	0.84	0.95	34.30	6.50	76%	4%	6.60	5.10	2.90	1.10	2.40	1.30	Diagnosis	48	no
'1997 Fazekas' [26]	1992	73	36%	6%	1.26	2.22	37.30		74%	5%	7.30		1.40		3.30		Diagnosis	24	no
'1998 PRISMS' [4]	1994	187	16%	3%	1.28		34.70	7.50	75%	3%	6.10	4.80	3.00	1.30	2.40	1.20	Diagnosis	24	no
'1998 Achiron' [23]	1995	20	0%	5%	1.61		33.80	2.40	80%	9%	3.95	2.86	1.55	0.76	2.82	1.65	Diagnosis	48	no
'2006 Polman' [22]	2001	315	46%	3%	0.73	1.27	36.70	7.80	67%	3%	4.30	4.80	1.50	0.77	2.30	1.20	Diagnosis	24	yes (2001)
'Origims'	2003	115	53%	5%	0.49	0.79	36.60	8.15	65%	4%	7.84	5.77	1.22	0.35	2.94	1.07	First Symptoms	24	yes (2001)
'2011 O'Connor' [28]	2004	363	46%	2%	0.54	0.68	38.40	9.00	76%	2%	8.6	7.1	1.4	0.7	2.68	1.34	First Symptoms	24	yes (2001)
'2010 Giovannoni'[34]	2005	437	61%	2%	0.33	0.53	38.70	9.90	66%	2%	8.90	7.40	1.35	0.60	2.90	1.30	Diagnosis	24	yes (2001)
'2010 Kappos' [1]	2006	418	46%	2%	0.40	0.73	37.20	8.60	71%	2%	8.10	6.40	1.40	0.70	2.50	1.30	First Symptoms	24	yes (2001)
'2011 Gold' [29]	2007	408	58%	2%	0.36		38.50	9.1	75%		5.8	5.78	1.3	0.67	2.48	1.24	Diagnosis		yes (2001)
'2012 Comi' [27], [30]	2007	556	52%	2%	0.30	0.38	38.50	9.1	66%		8.7	6.9	1.3	0.70	2.6	1.30	First Symptoms	48	yes (2005)

RRF  =  Rate of relapse free patients after 2 years, SE  =  Standard error, ARR  =  Annualized relapse rate over 2 years, SD  =  Standard deviation, 2001/2005 indicates the use of the original 2001 McDonald criteria or the 2005 revised criteria.

### Univariate and multivariate regression

**Figure 2:**
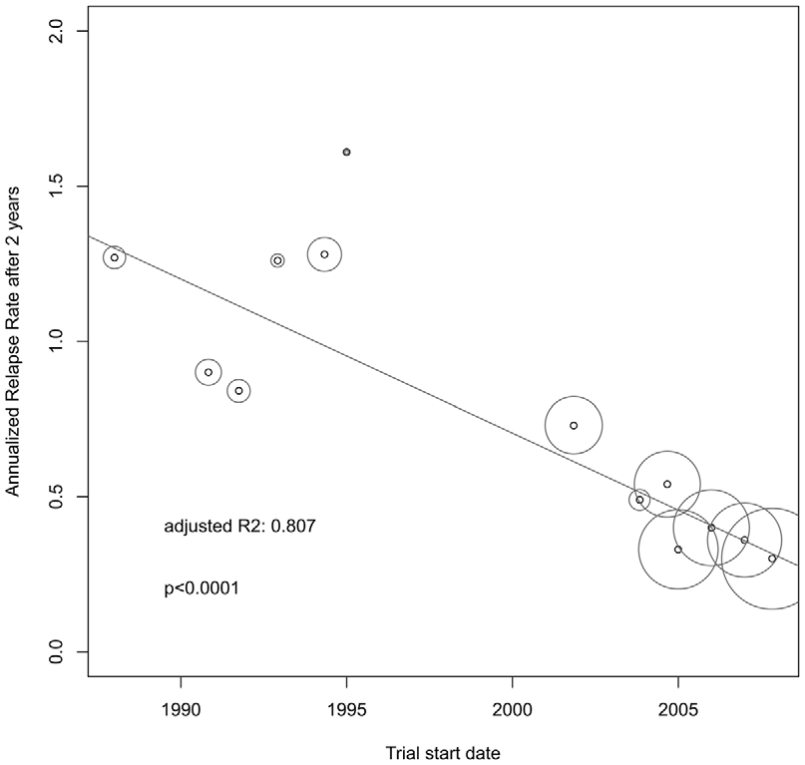
Change of annualized relapse rates of MS phase-3 placebo cohorts over time. n = 13. Circle size corresponds to weighting by patient numbers of each cohort. R2 is the proportion of variability in the data that is accounted for by the weighted univariate regression model.

**Figure 3:**
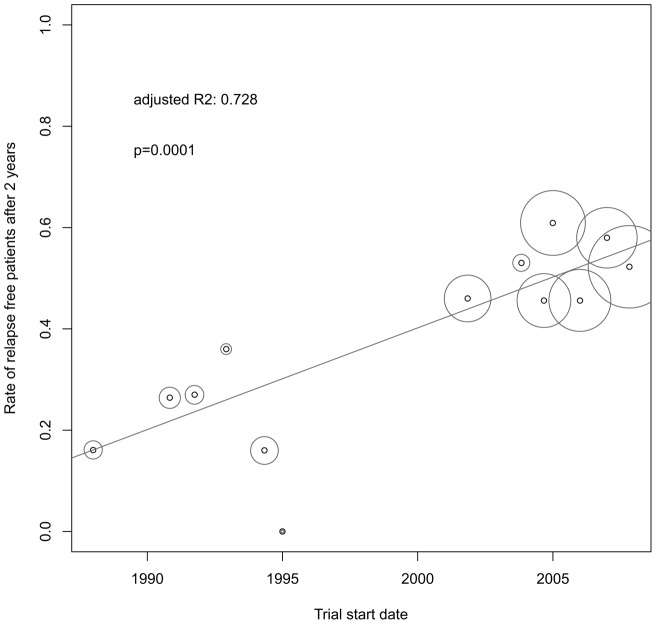
Change of the relapse free patients rate of MS phase-3 placebo cohorts over time. N = 13. Circle size corresponds to weighting by patient numbers of each cohort. R2 is the proportion of variability in the data that is accounted for by the weighted univariate regression model.

**Figure 4:**
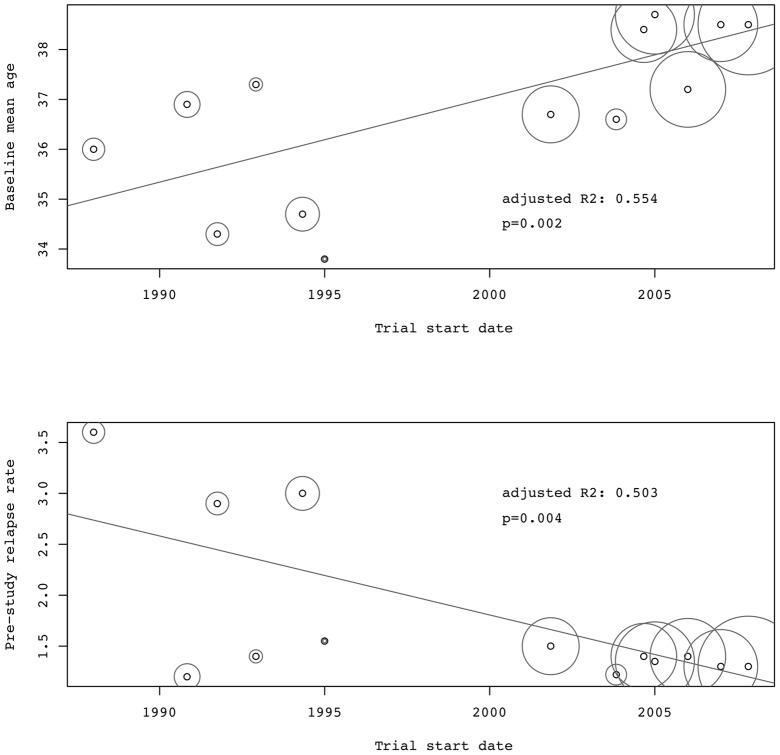
Change of mean age and pre-study relapse rates of MS phase-3 placebo cohorts over time. N = 13. Circle size corresponds to weighting by patient numbers of each cohort. R2 is the proportion of variability in the data that is accounted for by the weighted univariate regression model.

There was a significant reduction of the ARR over time (adjR^2^ = 0.807, p<0.0001, Figure 2) while, inversely, the RRF increased (adjR^2^ = 0.728, p = 0.0001, Figure 3) with a high association between both outcome measures (adjR^2^ = 0.815, p<0.0001). In the subgroup of studies not using McDonald criteria, i.e. before 2001, change over time was not significant. Univariate regression models showed a correlation of lower ARR and higher RRF with higher age, lower pre-study relapse rate and the use of the McDonald criteria. Definition of relapse duration was not significantly associated with the ARR but tended to show more relapses in the subgroup of studies using 48 hours as minimal relapse duration (data not shown). Further on, baseline mean age increased significantly over time while the pre-study relapse rate decreased significantly (Table 2 and Figure 4). By stepwise reverse exclusion of variables based on AIC we finally modelled a multivariate regression with baseline mean age and trial start dates as best predictors for the ARR (adjR^2^ = 0.821, p<0.0001). The RRF was best predicted by McDonald criteria and baseline mean age (adjR^2^ = 0.831, p<0.0001).

**Table 2:** Predictors for relapses - coefficient of determination in weighted regression models.

		ARR		RRF		Start Date	
		Coefficient estimate	adjusted r2	Coefficient estimate	adjusted r2	Coefficient estimate	adjusted r2
**Baseline Variables**	**Mean age**	−0.20 (*)	0.612	0.09 (*)	0.693	0.01 (*)	0.554
	**Rate females**	3.22	0.098	−1.52	0.143	0.01	0.034
	**Mean disease duration**	−0.11	0.117	0.05	0.096	0.01	-0.041
	**Pre-study mean relapse rate**	0.38 (*)	0.504	−0.17(*)	0.557	−0.01 (*)	0.503
	**Mean EDSS**	−0.15	0.010	0.16	−0.014	0.01	−0.014
**Definitions**	**Disease duration**	0.29	0.131	−0.05	−0.050		
	**Minimal relapse duration**	−0.01	−0.072	−0.01	−0.097		
	**McDonald criteria used**	−0.70(*)	0.744	0.30(*)	0.762		

Coefficient estimates and coefficient of determination (adjusted R2) of univariate regression models of baseline variables and outcomes. R2 is the proportion of variability in the data that is accounted for by univariate regression model. Variables were also analysed for their change over time (variables vs. start date). Significant (p<0.05 (*)) correlations were used for the multivariate modelling. RRF  =  Rate of relapse free patients after 2 years, ARR  =  Annualized relapse rate over 2 years.

### Random-effect meta-regression

In a second step we performed the random-effect meta-analysis. Models without predictive variables showed that the variance of outcomes alone could not explain the between-study variability. For ARR we calculated as follows: I^2^ = 98.9% (CI 97.8–99.7%) of total variability due to heterogeneity (tau^2^ = 0.16, p<0.0001). The corresponding value for the RRF was I^2^ = 97.4% (CI 95.0–99.1%, tau^2^ = 0.03, p<0.0001). No outliers could be detected by the “leave one out” method. Using start dates, baseline variables as well as the categorical definitions as single variables in each mixed-effect model for ARR and RRF, we found trial start dates, baseline mean age, rate of females, pre-study relapse rate and the use of McDonald criteria associated with both outcomes. Other variables failed to reduce tau^2^ significantly showing that there is no association with the outcomes in our analyses. Baseline mean EDSS, the definition of relapses and definition of disease duration even increased tau^2^ in some models. Results of all models calculated and the association of variables with outcomes, measured by their ability in reducing tau^2^, are presented in table 3. Models with two variables did not show a significant higher association with relapse outcomes than the model with the best single predictor, which were trial start dates for both outcomes ARR and RRF.

**Table 3:** Predictors for relapses – Association of variables with outcomes within a mixed-effect model.

		ARR				RRF			
Variables		Coefficient Estimate	p	taû2 (CI)	Reduction of taû2 in %	Coefficient Estimate	p	taû2 (CI)	Reduction of taû2 in %
**None (random-effect model)**				0.16 (0.08–0.50)				0.03 (0.02–0.09)	
**Start date**		−0.01	<0.0001	0.02 (0.01–0.15)	85.9	0.01	<0.0001	0.01 (0.01–0.04)	75.0
**Baseline Variables**	**Mean age**	−0.20	<0.0001	0.06 (0.03–0.22)	64.1	0.10	<0.0001	0.01 (0.00–0.03)	79.3
	**Rate females**	5.09	0.03	0.12 (0.06–0.37)	22.6	−2.61	0.002	0.02 (0.01–0.06)	47.9
	**Mean disease duration**	−0.13	0.1	0.14 (0.05–0.65)	12.7	0.07	0.04	0.02 (0.01–0.13)	26.9
	**Pre-study** **mean relapse** **rate**	0.30	0.004	0.08 (0.04–0.41)	47.4	−0.14	0.008	0.02 (0.01–0.07)	42.5
	**Mean EDSS**	0.20	0.6	0.17 (0.08–0.56)	−9.9	0.04	0.8	0.03 (0.02–0.11)	8.3
**Definitions**	**Disease duration**	0.47	0.02	0.12 (0.05–0.39)	26.3	−0.17	0.08	0.025 (0.014–0.09)	26
	**Minimal** **relapse** **duration**	0.01	0.75	0.18 (0.08–0.61)	−12.0	−0.01	0.2	0.03 (0.02–0.10)	22.4
	**McDonald criteria used**	−0.68	<0.0001	0.03 (0.01–0.12)	81.9	0.32	<0.0001	0.01 (0.00–0.02)	84.2

Taû2 is the estimate of residual heterogeneity. A higher reduction of taû2 within the mixed-effect models indicate a higher association with outcome. Testing for residual heterogeneity was significant with p<0.0001 in all cases. RRF  =  Rate of relapse free patients after 2 years, ARR  =  Annualized relapse rate over 2 years, CI  =  Confidence interval.

### SLC database

In the third step of our analysis plan the regression models were evaluated based on 505 pooled placebo patients of the SLC database with a follow-up of at least 2 years. Due to blinding reasons data from patients entering the trials after 1999 could not be included. Descriptive statistics are presented in table 4 and did not show obvious deviation from other RRMS populations. The list of variables included in the multivariate models was reduced compared to the models applied to the cohorts from the literature for the following reasons: (1) definition of relapse was not available; (2) definition of disease duration was always the same (since diagnosis) and (3) McDonald criteria could not be analysed as all patients had clinically definite MS. Within the Poisson regression model the pre-study relapse rate in the year before entry (coefficient estimate  = 0.12, p<0.001) could be identified as significant and best predictor of the ARR besides the date of entry in trial by year (coefficient estimate  =  −0.05, p<0.001). Analysing the RRF lead to the same results and showed an interestingly rapid decrease in the risk of relapses in studies after 1994 as indicated by the coefficients (Table 5). Baseline disease duration, age and gender had no further predictive value within multivariate models.

**Table 4:** Descriptive statistics of the individual patient cohort (SLC database).

N = 505	Mean (SD) N (%)	Median	Range
**Gender (female)**	366 (72.5%)	-	-
**Baseline disease duration** **in years**	7.2 (6.1)	5.5	0.3–37.7
**Baseline Age**	35.6 (8.1)	36	17–62
**Pre-study ARR last 12** **month**	1.4 (1.0)	1	0–6
**Pre-study ARR last 24** **month**	3.0 (1.6)	3	0–12

SD  =  Standard deviation.

**Table 5:** Individual patient cohort – final Poisson and logistic regression models.

N = 505		Outcomes				
		ARR		Relapse free after 2 years		
Regression Model		Poisson		Logistic (trial entry date as categorical variable)		
		Coefficient	P value	categories	coefficients	P value
**Variables**	**Trial entry date**	−0.05	**0.02**	1991–93	−0.03	0.9
				1994	0.13	0.8
				1995	−1.05	0.02
				1996	−2.67	<0.0001
				1997	−1.04	0.01
				1999	−1.39	0.04
	**Pre-study relapse** **rate (last year)**	**0.12**	**<0.0001**		0.16	**0.04**

ARR  =  Annualized relapse rate over 2 years, Logistic regression: Outcome was coded as 1 for at least 1 relapse in 2 years or 0 for no relapse in 2 years, reference category for trial entry was 1990 or earlier.

## Discussion

Our results of the yet largest meta-analysis of placebo cohorts in MS phase-3 trials confirm the notion of a reduction of relapse rates from 1990 to 2012 published studies. Individual data models from the SLC database between 1980 and 1999 also supported those findings. The most reliable meta-analytic results can be achieved by the analysis of pooled individual patient data as simulation studies indicated.[9] Interestingly our re-evaluation of the SLC data including the moderator “entry to trial date” lead to a final multivariate regression model without the pre-study disease duration as independent predictor. This extended the analysis of Held et al. from 2005 whose analysis did not consider “entry to trial date” and whose final model included the pre-study relapse rate and the pre-study disease duration.[5] In fact, the actual analysis demonstrates that the date of study entry has a higher predictive value for on-study relapse rates than the pre-study disease duration.

Mixed-effect models applied on the 12 placebo cohorts from the literature and the 13^th^ study from the SLC database suggested the McDonald criteria, baseline mean age and pre-study relapse rate as well as the rate of females as predictors for the on-study relapse activity. These models are more robust than simple regressions as mixed-effects models take unavoidable differences between the included trials into account.[10], [11] In our study, the congruence between the analysis of both relapse outcomes was higher in the mixed-effect models than in simple regression models.

The mixed-effect models showed a clear association between less relapses and the inauguration of the McDonald criteria that could be shown for the first time in a meta-analytic approach. Unfortunately, we could not test this finding on an individual case data base. To ensure blinding of individual placebo cohorts of RCTs only studies before 2000 could be considered in this analysis. This led to a data pool lacking RRMS cohorts diagnosed with McDonald criteria and a discrepancy between the study populations used for the meta-regression and the individual case data analyses. In how far the observed effects are based mainly on the fact that the use of McDonald criteria might be understood as a categorical time variable differentiating between older and younger studies, cannot be clarified based on the available data. Start dates and the use of McDonald criteria showed nearly the same predictive value in classic and random-effect regression models. Models with both variables did not further increase the predictive value of the univariate models. All other variables failed to neutralize these high correlations. A differentiation between a continuous or stepwise decrease of the ARR cannot be performed with the available data and prohibits estimating the ARR for future trials.

The correlation between new diagnostic criteria and disease activity refers to two major problems. First, defining more and more patients with a chronic-inflammatory but benign condition as MS patients leads to a further reduction of relapse rates in the continuously expanding MS population. This phenomenon was described by Sormani et al. as Will Rogers phenomenon in an cohort of 309 CIS patients in 2008.[31] The increasingly diagnosed so-called ‘radiological isolated syndrome’ (RIS) highlights this problem as real relapse activity is already known to be rare in this group. Within an US RIS cohort of 41 MS-symptom-free patients with a MRI suggestive of MS only 59% showed MRI activity and only 10 patients (25%) developed at least a clinically isolated syndrome (CIS) after a median of 5.4 years.[32] Secondly, results from treatment studies and prognostic factors within natural cohorts from the pre-McDonald era cannot be transferred to contemporary MS-cohorts. Therefore it is more pressing to find new prognostic criteria for disability than more sensitive, sub-clinical diagnostic criteria. Expanding the MS population towards RIS with more sensitive diagnostic criteria disseminates the MS stigma with a vague benefit for patients and physicians being highly ambivalent to recommend treatments at this stage.

The pre-study relapse rate was less predictive than McDonald criteria in the mixed-effect models. This may at least partially be affected by recruitment strategies between older and newer studies: In consequence of accepting uncertain or unspecific symptoms in the disease history to fulfil a required number of relapses for study inclusion the true pre-study relapse rate may be overestimated in the newer studies. Probably a stricter definition of pre-study relapses e.g. steroid treatment, documented motor, visual or brainstem symptoms might be useful to have a more robust estimate for historical relapse frequencies.

Besides the relevance of pre-study relapses, McDonald criteria and trial start dates in our mixed-effect models give additional statistically reliable and clinically meaningful results: Young female patients have a higher short-term risk for further relapses.

The correlation of age and on-study relapses within the literature cohorts confirm the analysis of Inusah et al. but stays in contrast to the SLC data, where age did not increase the predictive value of multivariate models including pre-study relapses and year of study entry. This finding might be affected by the lower mean age of the SLC patients. As in the case of McDonald criteria, the restricted availability of individual case data denied a clarification of this discrepancy. Possible explanations might be higher sensitivity of McDonald criteria in older patients with lower relapse activity or previous treatment with approved drugs. In addition, the availability of approved therapies since 1993 may have lead to a selection towards older and less active patients for studies. This might be as well the reason for the nearly stepwise decrease of the relapse risk after 1994 observed within the logistic regression of the individual case data. Future cohorts with younger patients might help to clarify the relevance of age and disease duration for onstudy disease activity. The finding by Inusah et al., that different definition of relapses by minimal duration (24 or 48 hours) influences the ARR, could not be confirmed by our data. A small effect may be missed by our reduced data set, as only 4 studies used the 48 hours definition. [8] But, as our data even show a tendency towards more relapses in the subgroup with the more rigorous definition, we do not identify real implications for future trial design in this.

According to our findings future RRMS trials need a careful definition of inclusion criteria and novel trial designs have to be discussed as solution for an unpredictable ARR. Recently, Nicholas et al. published simulation studies that proposed adaptive designs to control sample sizes and power.[33].

Meta-analyses are always restricted by the available studies. Only 12 phase-3 RCTs in RRMS with a follow-up of at least 18 months have been published in the last 20 years. Negative MS trials showing no effect of an investigational drug are difficult to retrieve. In fact, low event rates in placebo groups might be a major reason for not showing treatment effects. This was the case of the ORIGIMS trial, which we could include in our analysis. As our systematic literature research did not reveal one single negative trial, a substantial publication bias must be stated. Therefore our data set probably even overestimates the true ARR. The low number of studies restricted our mixed-effect models to a maximum of two moderators per model and inhibited a direct and statistically meaningful comparison of different moderators. Due to missing data the MRI criteria and prior treatments could not be included in our analyses but might have impacted on the disease activity and therefore relapse rates as well.

## Conclusions

Up to now all analysed predictors failed to satisfactorily explain the lowering of relapse rates in phase-3 trials over the last decades. Inclusion criteria for future phase-3 trials in RRMS with relapse-based outcomes should be adjusted based on the results of our analysis showing that higher age and lower pre-study relapse rates are associated with a lower on-study ARR. The relevance of prior disease modifying treatments, the change of relapse rates over time and even more important the effect of new diagnostic criteria must be further analysed on an individual case data base. We emphasize the importance to continue gathering and sharing the data of placebo cohorts – and treatment data – as it has been started in the SLC in 2001.

## Supporting Information

Checklist S1 **PRISMA Checklist.**
(DOC)
